# Parents’ attitudes towards the management and prevention of diarrhoea in children under five in Namibia

**DOI:** 10.4102/hsag.v30i0.2714

**Published:** 2025-05-27

**Authors:** Maria F. Bauleth, Honore K. Mitonga, Lusia N. Pinehas

**Affiliations:** 1 School of Nursing and Public Health, Faculty of Health Sciences and Veterinary Medicine, University of Namibia, Oshakati, Namibia

**Keywords:** diarrhoea, under-five children, prevention, child health, attitudes

## Abstract

**Background:**

Every year, nearly 1.7 billion cases of childhood diarrhoea and around 525000 deaths among under-five children are reported globally. Information about parents’ or caregiver’s attitude concerning child health is crucial for improving infant and child wellbeing in the community.

**Aim:**

The aim of the study was to conclude the level of parents’ or caregivers’ attitude on managing and preventing diarrhoeal disease among under-five children in the Engela district.

**Setting:**

The Engela district forms part of the seven constituencies in the Ohangwena region. More than half (62.3%) of the Ohangwena population lives in Engela.

**Methods:**

A cross-sectional, descriptive study was conducted. Furthermore, 530 parents or caregivers participated in the study. A questionnaire was administered through face-to-face interviews. Factors associated with negative attitudes were determined by performing logistic regression.

**Results:**

Overall, 530 parents or caregivers participated in this study. Diarrhoea prevalence among the children under five was 24%. Factors such as parent or caregiver age group, residential area, possession of radio and education level were significantly associated with attitude (*p* < 0.05). The strongest predictor of attitude was being a resident of an informal settlement (odds ratio of 21.9) and the parent or caregiver not being educated documenting an odds ratio of 15.8.

**Conclusion:**

In conclusion, a greater number (72%) of parents or caregivers were considered to have a negative attitude. Furthermore, 32.5% strongly agreed and 42% agreed that child’s parents-related factors contribute to child suffering from diarrhoeal disease.

**Contribution:**

Therefore, when planning for interventions, existing public health programmes targeting prevention and management of diarrhoea should consider age, residential area, level of education and motivation for ownership of radio, perceptions and beliefs of mothers or caregivers.

## Introduction

According to Padhy and Sethi (2017) to improve infant and child morbidity and mortality in the community, it is vital to have information about parents’ and carers’ attitudes towards child health. A trustworthy picture of health-seeking behaviours, homecare routines and the identification of diarrhoeal warning indicators can be obtained from the knowledge, attitudes and practices of parents and carers. The most frequent cause of death in cases of diarrhoeal illness is dehydration, which can be treated or avoided. Nonetheless, according to the research performed on diarrhoea prevention among parents of children under five in Northwest Ethiopia’s Enemay district, 27% of the parents argued that diarrhoea can be avoided (Agegnehu et al. 2019). Treatment with oral rehydration solution (ORS) is particularly useful in reducing deaths from this disease (Munos, Walker & Black 2010). Additionally, according to a study carried out in Dire Dawa, Eastern Ethiopia, more than half (55%) of the participants expressed disagreement with the at-home administration of ORS for the treatment of diarrhoeal illness in children under five (Workie, Sharifabdilahi & Addis 2018). Likewise, a sizeable portion of participants (61.4%) disagreed that treating diarrhoeal illness in children at home was a viable option. Approximately 51.5% of parents said their kids did not like the flavour of ORS (Workie et al. 2018). Moreover, there are other preventive measures like immunisation, sanitation, proper hygiene and breastfeeding. However, most developing nations face issues with insufficient food, water and personal cleanliness, unhygienic circumstances, rising food insecurity and unhealthy cultural beliefs and behaviours (Imoh 2013). Based on a study related to household water treatment methods carried out in Northwest Ethiopia, 45.2% of the partakers had bad attitude (George et al. 2014). In addition, parents’ or caregivers’ knowledge and attitude are reported to be to related with their culture, socio-demographic circumstances, exposure to health education and additional factors (Skoufias, Vinha & Conroy 2011).

According to Namibia Demographic and Health Survey (NDHS) in 2014, diarrhoeal disease is considered a public health problem in Namibia, with estimated prevalence of 17%. Nevertheless, diarrhoeal disease among children under five is estimated to be highest at 19% in the Ohangwena region, where the study was conducted (Mengistie, Berhane & Worku 2013; Ministry of Health and Social Services 2018).

In addition, social factors such as parents’ knowledge, attitude and practice related to preventing diarrhoea are reported to significantly influence child health and survival (Kier & Dai 2018; Taffa & Chepngeno 2005). Hence, the aim was to assess the level of parents’ or caregivers’ attitudes towards managing and preventing diarrhoeal disease among children under five in the study area. The main variables on attitude included attitude-related causes of diarrhoea, attitude related to treatment of diarrhoea and attitude related to the prevention of diarrhoea.

## Research methods and design

### Study design

A cross-sectional, descriptive study was carried out to collect data from partakers in the study area between 17 January 2019 and 09 March 2019.

### Study setting

Engela district in the Ohangwena region is situated in the northern part of Namibia, ±738 km from the capital city Windhoek, sharing borders with the southern part of Angola (Ministry of Health and Social Services 2018). The Ohangwena region has an estimated population of 281 358 of which 62.3% reside in the study area (Engela district). The district comprises a regional hospital, two health centres and 18 clinics. Nonetheless, Engela district is prone to natural hazards such as drought and flood that subsequently displace the communities and predispose them to famine and waterborne diseases. During dry season, some areas are sandy and do not have proper road infrastructures, therefore they can only be accessed with 4 × 4 vehicles. Consequently, this affects service delivery such as on-time supply of essential equipment from health sectors as well as other stakeholders such as Ministry of Health and Social Services as well as regional and district authorities (Chopra et al. 2013; Thiam et al. 2017). These challenges hinder the provision of quality health services by all stakeholders involved.

### Study population

The population of the current study included parents or caregivers (women and men) from households with under-five children aged between 1 and 59 months and who are inhabitants in the Engela district for a period of at least 1 year.

### Sample size

In the current study, Cochran’s formula was used to determine the sample size, $n=z^{2*}\frac{p*q}{e^{2}}$ (Association, G.U. Mathematics 2013), where *n* is the sample size, *z* is the desired confidence level 95%, *p* is the expected prevalence, *q* is the expected non-prevalence (1-*p*) and *e* is the relative desired precision. $n=\frac{1.96^{2*}0.5*0.5}{0.05^{2}}=384.16$ households (*HH*) = 384 HH. The final sample size together with 10% of non-respondent rate is 38 + 384 = minimum 422 HH, nevertheless, we managed to survey 530 houses.

### Sampling procedures

The study employed the multistage cluster sampling method, with constituencies being regarded as clusters. The sample procedure was carried out as indicated in Table 1. In the initial round, five of the seven constituencies were chosen at random. All seven constituencies were listed and given numbers to conduct a basic random sample. After that, a number that was included in the study was chosen at random by the researcher. Proportionate sampling was employed in the second phase to choose the villages from the constituency. A total number of 131 villages were randomly selected from the following constituencies: Ongenga (38), Oshikango (14), Endola (34), Engela (29) and Ondobe (16). In addition, households were selected using a systematic sampling method by randomly generating numbers using a scientific calculator and then selecting every fifth household from all five constituencies.

**TABLE 1:** Multistage sampled constituency villages and households.

Name of selected constituency	Number of villages or constituencies	Proportion of villages per constituency Weight %	Total selected villages	Number of HH or constituency	Proportion of HH per village Weight %	Total selected HH
Ongenga	75	29	38	3887	17	90
Oshikango	29	11	14	5378	23.7	126
Endola	69	26	34	4654	20.5	109
Engela	57	22	29	4903	21.6	115
Ondobe	32	12	16	3851	16.9	90

**Total**	**262**	**100**	**131**	**22 673**	**100**	**530**

Note: Total number for constituencies =7.

HH, households.

However, only household with an under-five child was qualified for the study and one participant was selected per household to take part in the study. Simple random sampling was done to select parents or caregivers who participated in the study.

### Data collection tool and method

A structured researcher-administered questionnaire was developed based on the reviewed literature. In addition, the section containing the household characteristics was taken from NDHS (2014).

When gathering data, the researcher together with the student nurses and health extension workers (HEWs) narrated the questions to the respondents, recorded their answers precisely as provided by the respondents and after completion, the researcher left with the questionnaire (Weaver et al. 2010; World Health Organization 2018). Furthermore, the questionnaire comprised free sections: section A: socio-demographic, section B: household characteristics and section C: aspects related to attitude. The questionnaire comprised closed-ended questions. In addition, the questionnaire comprises variables related to perceived causes of diarrhoea, treatment and prevention of diarrhoea. On the other hand, socio-demographic factors included exposure variables such as parent or caregiver’s age, gender, educational status, residential area, employment status, average monthly income and ownership of radio and television.

Attitude analysis was quantified and categorised as positive or negative. The attitude significant variables included perceptions on causes of diarrhoea (6 points), treatment of diarrhoea (4 points) and prevention of diarrhoea (5 points). A 5-point Likert scale (strongly agree, agree, neither agree nor disagree, disagree and strongly disagree) comprising 15 points was used to assess parents’ or caregivers’ attitudes regarding diarrhoeal disease. From the 15 points, six questions assessed negative attitude, while eight questions assessed positive attitude. Relevant answers were assigned a score of 1 and irrelevant answers a score of 0 (Table 2). Each respondent who obtained 70% and above was considered to have positive attitude and those who scored below 70% were categorised as having negative attitude.

**TABLE 2:** Logistic regression – factors associated with diarrhoea in children.

Variable	Wald	*df*	Sig.	Exp(B)**	95% CI for Exp(B)	
					Lower	Upper
**Caregivers’ age group (years)**	5.29	4	0.26	-	-	-
18–30	1.05	1	0.31	1.59	0.65	3.88
31–40	1.00	1	0.32	1.56	0.65	3.75
41–50	0.86	1	0.35	1.47	0.64	3.44
51–60	4.77	1	0.03*	2.92	1.12	7.61
Availability of radio	4.20	1	0.04*	1.72	1.02	2.89
Residential area	16.60	2	0.00*	-	-	-
Informal settlement	6.70	1	0.01*	21.92	2.12	227.28
Rural areas	0.30	1	0.59	1.73	0.24	12.45
Gender (1)	0.43	1	0.51	1.36	0.55	3.35
**Religion**	1.40	4	0.85	-	-	-
Protestant	0.92	1	0.34	1.88	0.52	6.86
Catholic	0.30	1	0.59	1.46	0.38	5.67
Anglican	0.57	1	0.45	1.66	0.45	6.15
Others	0.00	1	1.00	0.000	0.00	-
**Education level of caretaker and/or mother**	11.36	3	0.01*	-	-	-
Not educated	5.66	1	0.01*	15.77	1.63	152.90
Primary education	3.41	1	0.07	8.15	0.88	75.63
Secondary education	2.52	1	0.11	6.12	0.65	57.25

CI, confidence interval; *df*, degrees of freedom; Sig., significance; Exp(B),Exponentiation of the B coefficient.

* , *p*-value statistically significant at 0.05;

** , Exp(B) – Odd ratios.

The data collection process has been monitored by the main researcher daily, while the principal investigators inspected the completeness of the questionnaire.

### Data quality control

The questionnaire was taken from the literature and adjusted with consideration for the local context to ensure its validity. Next, a pilot study was conducted in the constituency that was excluded from the study using 5% of the sample size. In addition, the instrument was adjusted in accordance with the pilot study’s results. A Cronbach’s alpha calculation of 0.810 indicated that the data collection items measured the same idea, supporting the reliability and validity of the tool.

Before data collection, the main researcher trained the student nurses and HEWs on data collection.

### Data analysis

For data analysis, the researcher used International Business Machines (IBM) Statistical Package for Social Science (SPSS) software version 24. The descriptive statistics was used to summarise the study results and inferential statistics was used to make inferences and draw conclusions after analysing data. To summarise the data, descriptive statistics were used, which were presented as frequency distribution tables, consisting of frequencies, percentages and 95% confidence intervals. The levels of attitude were related to socio-demographic characteristics based on the 5-point Likert scale consisting of the following points – (1) Strongly Disagree, (2) Disagree, (3) Neither Agree nor Disagree, (4) Agree and (5) Strongly Agree. Attitude was assessed based on attitude related to causes of diarrhoea, treatment of diarrhoea and prevention of diarrhoea. Attitude related to prevention of diarrhoea comprised six questions, of which the first four questions (Witchcraft and evil eye cause diarrhoea, a child can develop diarrhoea as a result of maternal and parental factors, teething causes diarrhoea and breastfeeding, while pregnancy causes diarrhoea) are worded negatively, and the last two questions (human faeces can contribute to diarrhoeal disease and diarrhoea is common among bottle-fed children) are worded positively. In addition, four questions assessed attitude towards the treatment of diarrhoea whereby the first two questions on the list are worded positively and the last two are worded negatively. Lastly, the last section of the questions assessed aspects related to diarrhoea prevention comprising five questions of which the first four questions are worded positively and the last question is worded negatively.

For the acceptable positive answers that reflected positive attitude, a correct answer was allocated 5 points and wrong answers were allocated 0 points. Furthermore, a summation score for each respondent was derived by adding their responses for all the questions and converted into percentages. The modified Bloom’s cut-off points were adopted to categorise attitude, with scores between 70% and 100% considered positive and scores below 70% considered negative. The overall respondents’ attitude was categorised into positive attitude for scores between 70% and 100% and negative attitude for scores less than 70%. In addition, the Chi-square (*Χ*^2^) test was employed to test for statistical relationship between attitude and socio-demographic characteristics analysed at the bivariate level. In addition, bivariate analysis was used to determine associations between levels of attitudes and the socio-demographic characteristics. In addition, demographic characteristics with a *p*-value less than 0.5 were further analysed using binary logistic regression with the output presented as odds ratios. For all statistical procedures, a 95% confidence interval was set with a margin of error or a level of significance of 0.05. In the current study, the outcome variable was the attitude of the parents or caregivers on diarrhoeal disease.

### Ethical considerations

Ethical clearance to conduct this study was obtained from the University of Namibia Research Ethics Committee (Reference no: SON/533/2019).

## Results

### Attitude of parents or mothers on acute diarrhoea

Table 3 presents attitude-related questions and distribution of scores attained by parents or caregivers in relation to prevention, contributing factors or causes and treatment of diarrhoeal disease in children under five. Regarding the contributions or causes of the development of diarrhoea disease: Almost half of the respondents (43%) of the parents or caregivers agreed that witchcraft and evil eye can cause diarrhoea. Similarly, a large proportion (42%) agreed that parental and maternal factors can lead to the development of diarrhoea in children under five (believing that skin tag at the parents’ perineal area can contribute to a child developing diarrhoea).

**TABLE 3:** Attitude questions related to diarrhoea disease.

Variables	Strongly agree		Agree		Neither agree nor disagree		Disagree		Strongly disagree		Total percentage of negative attitude (%)
	Frequency	%	Frequency	%	Frequency	%	Frequency	%	Frequency	%	
**Attitudes towards causes of diarrhoea**											
Witchcraft and evil eye cause diarrhoea	228	43.0	129	33.8	12	2.3	58	10.9	48	9.1	79.1
Child can develop diarrhoea as a result of maternal and parental factors (*eemhalo*)	172	32.5	223	42.1	12	2.3	63	11.9	60	11.3	79.9
Teething causes diarrhoea	225	42.5	262	49.4	17	3.2	19	3.6	7	1.3	95.1
Breastfeeding while pregnant causes diarrhoea	211	39.8	237	44.7	15	2.8	41	7.7	26	4.9	87.3
Human faeces can contribute to diarrhoeal disease	195	36.8	249	47.0	13	2.5	51	9.6	22	4.2	16.3
Diarrhoea is common among bottle-fed children	186	35.2	210	39.6	15	2.8	78	14.7	41	7.7	25.2
**Attitude toward diarrhoea management**											
Chronic diarrhoea in children is treated with Western medicine	126	23.8	167	31.5	28	5.3	103	19.4	106	20.0	44.7
It is essential to continue breastfeeding when a child has diarrhoea	228	43.0	179	33.8	17	3.2	58	10.9	48	9.1	23.2
Prayers are powerful and can cure child with diarrhoea	63	11.9	85	16.0	14	2.6	159	30.0	209	39.4	30.5
Liquid food aggravates diarrhoea	128	24.2	178	33.6	17	3.2	119	22.5	88	16.6	61
**Attitude towards prevention**											
Diarrhoea can be prevented through immunisation and/or vaccination	167	31.5	243	45.8	17	3.2	72	13.6	31	5.8	22.6
Hand washing with soap prevents diarrhoea	286	54.0	214	40.4	13	2.5	7	1.3	10	1.4	5.2
Child’s or infant faeces are hazardous to health	138	26.0	172	32.5	22	4.2	111	20.9	87	16.4	41.5
Diarrhoea is a problem in the community	214	40.4	219	41.3	18	3.4	50	9.4	29	5.5	18.3
Hand washing should be performed only when adequate water is available	60	11.3	75	14.2	18	3.4	205	38.7	172	32.5	43.8

Note: *Eemhalo*: a word in Oshiwambo language used to describe skin lesions at the mother’s perinium believed to cause diarrhoea in under-five children.

However, 44.7% of respondents believed that breastfeeding during pregnancy causes diarrhoea, and 49.4% agreed that teething causes it. Nevertheless, 47% of those surveyed concurred that diarrhoea is caused by human excrement. In addition, 39.6% of respondents agreed that bottle-fed children are mostly affected by diarrhoea.

Management of diarrhoeal illness in under-five children: 20% of respondents strongly disagree that Western medicine is the best way to treat chronic diarrhoea in children. Nonetheless, 43% of participants firmly concur that it is critical to keep nursing a child who is experiencing diarrhoea.

However, 16% of the participants concurred that prayers could heal a child suffering from diarrhoea, and 11.9% strongly agreed that prayers may heal diarrhoeal illness. Furthermore, 33.6% of respondents concurred that diarrhoea is made worse by eating liquid meals.

Regarding the prevention of diarrhoeal disease, most respondents (45.8%) agree and 31.5% strongly agree that immunisation can protect a child from developing diarrhoea. Likewise, 54% of the respondents strongly agree that diarrhoea can be prevented by washing hands with soap and water.

Furthermore, 14.2% of respondents agree and 11.3% strongly agree that one wash hands only when there is sufficient water available. Nevertheless, 20.9% disagree that infant or child faeces are unsafe for health. Finally, majority of the respondents (41.3%) agree and 40.4% strongly agree that diarrhoea in children under five is problematic in the community.

**FIGURE 1:**
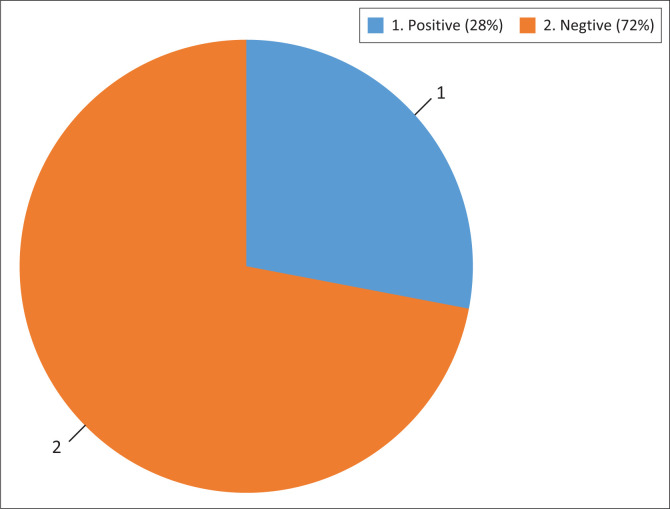
Parents’ or caregivers’ attitude towards diarrhoea in under-five children.

Approximately, 27.7% (*n* = 147) of respondents were classified as having positive attitude towards prevention, cause and treatment of diarrhoeal disease by not agreeing to questions that are worded as negative responses and agreeing to the questions that are worded as positive questions. Alternatively, most respondents (72.3%) (*n* = 383) were classified as having negative attitude by agreeing to questions that were worded as negative responses and disagreeing to questions that were worded as positive responses (Figure 1).

Table 3 presents the socio-demographic characteristics of the parents or caregivers of under-five year children. Overall, 530 parents or caregivers took part in the study. Mean age was 40 years (standard deviation [s.d.] = 8.38). More respondents (29.4%) (*n* = 156) were aged between 18 and 30 years, and 26.4% (*n* = 140) were between 31 and 40 years, respectively.

Similarly, the age group of 31–40 years was reported to have higher negative attitude at 74.3% (*n* = 113) followed closely by the age group of 18–31 years at 72.4% (*n* = 104). Nonetheless, the observed difference was statistically significant (*p* < 0.003). Majority of the respondents (94.3%) (*n* = 500) were women; nevertheless, the higher negative attitude at 80% (*n* = 24) was reported among men as compared to women at 71.8% (*n* = 359); however, the observed difference was not significant (*p* > 0.05). A significant number of respondents (79.8%, *n* = 383) were residing in rural areas; conversely, a large percentage (97.1%) (*n* = 99) of respondents categorised as having negative attitude were from informal settlement, and the detected difference was statistically significant (*p* < 0.05). The respondent’s education level was found to be low, only 1.9% (*n* = 10) attended high education and 16% (*n* = 85) of the respondents never attended school. Similarly, negative attitude is reported to be high at 78.8% (*n* = 67) among respondents who did not attend school; nonetheless, the observed difference was not significantly related to diarrhoea prevalence. A greater number of the participants (68.9%) (*n* = 365) were in possession of a radio. However, 84.5% (*n* = 139) of the participants who did not possess a radio were categorised as having negative attitude, and ownership of radio was significantly associated with the respondents’ attitude (*p* < 0.05). Prevalence of diarrhoea was 23.8% (*n* = 126), and parents or caregivers classified as having negative attitude reported the majority of diarrhoea prevalence among children under five (78.6%, *n* = 99). However, observed difference was not statistically significant. Under-five children were undernourished 66.8% (*n* = 354); however, undernutrition was not significantly associated with parent or caregiver attitude towards diarrhoea disease. Slightly more than half of the respondents were Protestants (53.8%) (*n* = 285). Similarly, the majority of the parents or caregivers whose religion was protestant (51.2%, *n* = 196) were classified as having negative attitude. Nevertheless, the observed difference was not significantly related to diarrhoea prevalence.

### Factors influencing the parents’ or caregivers’ attitude

Logistic regression analysis of factors associated with mother’s attitude towards diarrhoeal disease among the children under five in the study area was performed to assess the probability of factors that have an association with the occurrence of diarrhoea. The model comprising all the predictors was statistically significant, χ^2^ (20, *N* = 530) = 75.89, *p* ˂ 0.001, indicating that the model was able to differentiate among parents or caregivers with positive attitude and those with negative attitude. The model as a whole explained between 13.3% (Cox and Sell R square) and 19.3% Nagelkerke R-squared) of the variance in attitude and properly classified 72.5% of the cases. Based on the results shown in Table 4, four of the independent variables made an exclusive statistically significant contribution to the model (parents or caregiver residential area, ownership of radio, age group and parent or caregiver education level). Parent or caregiver residential area (informal settlement) was found to be the strongest predictor of attitude recording an odds ratio of 21.92. This indicates that parents or caregivers who reside in informal settlements were 21 times more likely to have a negative attitude as compared to those who are from rural areas. In addition, the level of education recorded an odds ratio of 15.77, indicating that participants with no education were 15 times more likely to have negative attitude as compared to other participants.

**TABLE 4:** Parent or caregiver socio-demographic characteristics in relation to attitude scores.

Variable	Negative attitude		Positive attitude		Total		*p* *
	*n*	%	*n*	%	*n*	%	
**Parent or caregiver age (years)**							0.003*
18–30	113	72.4	43	27.6	156	29.4	-
31–40	104	74.3	36	25.7	140	26.4	-
41–50	87	69.6	38	30.4	125	23.6	-
51–60	50	79.4	13	20.6	63	11.9	-
≥ 60	29	63.0	17	37.0	46	8.7	-
**Parent or caregiver gender**							0.330
Male	24	80.0	6	20.0	30	5.7	-
Female	359	71.8	141	28.2	500	94.3	-
**Area of residence**							0.001*
Urban	3	60.0	2	40.0	5	0.9	-
Informal settlement	99	97.1	3	2.9	102	19.2	-
Rural areas	281	66.4	142	33.6	383	79.8	-
**Parent or caregiver education level**							0.008*
Not educated	67	78.8	18	21.2	85	16.0	-
Primary education	177	72.0	69	28.0	246	46.4	-
Secondary education	132	69.8	57	30.2	189	35.7	-
Higher education	7	70.0	3	30.0	10	1.9	-
**Ownership of radio**							0.001*
Yes	244	66.8	121	33.2	365	68.9	-
No	139	84.2	26	15.2	165	31.1	-
**Prevalence of diarrhoea**							0.076
Yes	99	78.6	27	21.4	126	23.8	-
No	284	70.3	120	29.7	404	76.2	-
**Prevalence of malnutrition**							0.708
Undernutrition	254	71.8	100	28.2	354	66.8	-
No undernutrition	129	73.3	47	26.7	176	32.2	-
**Religion**							0.241
Protestant	196	51.2	89	60.5	285	53.8	-
Catholic	57	14.9	16	10.9	73	13.8	-
Anglican	117	30.5	39	26.5	156	29.4	-
Other	13	3.4	3	2.0	16	3.0	-
**Monthly income** (N$)							0.491
± 200.00–2000.00	364	73.7	130	26.3	494	93.2	-
> 2000.00–5000.00	14	53.8	12	46.2	62	4.9	-
> 5000.00–10000.00	1	20.0	4	80.0	5	0.9	-
> 10000.00	4	80.0	1	20.0	5	0.9	-

**TOTAL**	**383**	**72.3**	**147**	**27.7**	**530**	**100.0**	**-**

* , *p*-value Pearson Chi-square statistically significant 0.05.

## Discussion

Results from the current study displayed that the majority (72%) had a negative attitude related to causes, treatment and prevention of diarrhoea. The outcomes are consistent with the findings from a study conducted in rural Maharashtra among school-going children and their parents that reported attitude and practice regarding diarrhoeal disease to be very poor (Promtussananon & Peltzer 2003). With regard to contributions to the development of diarrhoea disease, most respondents believed that diarrhoea can occur as a result of witchcraft and evil eye and by maternal and parental factors *(a shared belief in the study area that the skin tag at parents’ perineal area can contribute to the development of diarrhoea among under-five children if not removed)*, and this is consistent with a study by Ansari et al. (2012), which based on the focus group discussions found that witchcraft and teething were found to be the leading cause of diarrhoea in the villages. However, it has been reported that a traditional approach to the management of diarrhoea, for instance, the practice of cutting mothers in the vaginal and anal areas in Namibia, has no positive effects either on the child or the mother’s inspiration to seek medical treatment (Das et al. 2013). Nevertheless, some participants believed that teething causes diarrhoea equally, and a significant number believed that breastfeeding when pregnant leads to diarrhoea. According to a study conducted in Khartoum, Sudan in the emergency paediatric hospital, it has been reported that 95.7% of mothers thought that teething causes diarrhoea (Thabit 2019). Similarly, based on a study carried out in Southwest Ethiopia on prevalence and factors associated with stunting, stopping breastfeeding and inappropriate feeding were found to be significantly related to stunting (Taffa & Chepngeno 2005). In addition, 36.8% strongly agreed and 47% agreed that human faeces contribute to diarrhoea prevalence. Similarly, 35.2% strongly agreed and 39.6% agreed that diarrhoea occurs mostly among bottle-fed children.

Management of diarrhoeal disease in children under five: 20% of the respondents strongly disagree that chronic diarrhoea in children can be managed with Western medicine. The findings conform with the findings from a study carried out at the emergency paediatric hospital in Khartoum, Sudan, where 30.9% of mothers reported to have used traditional methods and 16% gave self-remedies (Kier & Dai 2018). Conversely, 43% of respondents strongly agree that it is essential not to stop breastfeeding when a child has diarrhoea. Conversely, based on a study conducted in Mirzapur, rural Bangladesh, only 0.4% of the respondents indicated that breastfeeding is an effective measure to prevent childhood diarrhoea (Das et al. 2013).

This study also identified factors such as religion to cure diarrhoea, whereby some respondents indicated that prayers can cure a child who is suffering from diarrhoea. These findings are like the findings of a study conducted by Ansari et al. (2012) who reported factors such as supernatural to be regarded as the cause of diarrhoea disease. In addition, 33.6% of respondents agreed that liquid food aggravates diarrhoea.

Relation with the prevention of diarrhoeal disease: A majority of respondents in the current study agree that immunisation prevents a child from developing diarrhoea. Similarly, a majority of the respondents strongly agree that hand-washing with soap and water prevents diarrhoea. Nevertheless, some respondents indicated that hands should be washed only when there is enough water available. The findings are dissimilar to the findings from a study carried out in Mirzapur, rural Bangladesh, where it has been reported that 49.5% and 7.7% indicated washing hands and proper disposal of human faeces to be effective measures for preventing childhood diarrhoea (Das et al. 2013). Lastly, the majority of the respondents agree diarrhoea prevalence in children under five to be a problem in their communities.

In this study, factors, such as monthly income, ownership of radio and residential area, were statistically significantly related to attitude. Mass media such as television and radio are stated to have been successfully used to regularly bring health-related messages to individuals and families for their awareness of the risks and prevention of diarrhoea (Weaver et al. 2010).

Based on logistic regression analysis shown in Table 4, factors, such as parent or caregiver education level, age group, possession of radio and residential area, were statistically significant at the significant level of 0.05. The strongest predictor for negative attitude was the informal settlement residential area recording an odds ratio of 21.92 together with parents or caregivers not being educated, recording an odds ratio of 15.77. This indicates that parents or caregivers residing in informal settlements were 21 times more likely to have a negative attitude as compared to those residing in rural areas. In addition, parents or caregivers with a lower level of education were 15 times more likely to have a negative attitude.

## Conclusion and recommendations

In conclusion, a large proportion of parents or caregivers were categorised as having negative attitude associated with causes, management and prevention of diarrhoea disease. Nevertheless, logistic regression shows factors such as age category, residential area, as well as possession of radio as independent predictors of attitude. Hence, a number of mothers need better attitudes to improve the prevention and management of diarrhoeal disease. Therefore, health education and behavioural change programmes to improve attitudes in these communities are needed. Evaluation of existing public health programmes on child health is needed to highlight gaps in implementation.
